# Post-Intubation Sore Throat and Menstruation Cycles

**DOI:** 10.5812/aapm.11416

**Published:** 2013-09-01

**Authors:** Amirali Orandi, Amirhossein Orandi, Atabak Najafi, Fatemeh Hajimohammadi, Sara Soleimani, Somayeh Zahabi

**Affiliations:** 1 Department of Anesthesia and Critical Care, Tehran University of Medical Sciences, Sina Hospital, Tehran, Iran; 2 Department of Anesthesia and Critical Care, Tehran University of Medical Sciences, Amiralam Hospital, Tehran, Iran

**Keywords:** Intubation, Laryngoscopy, Pharyngitis, Menstrual Cycle

## Abstract

**Background:**

Postoperative sore throat is one of the most common complications of general anesthesia and intubation with prevalence of 18%-65% in different studies. Several risk factors including female gender, postoperative nausea and vomiting and so on have been mentioned.

**Objectives:**

The aim of this study was to evaluate the incidence of postoperative sore throat in females and its association with menstrual cycles.

**Patients and Methods:**

One hundred females between 18-45 years old with ASA class I or II without predicted difficult airway that were candidate for operation in supine position were enrolled in study. Patients who had pulmonary disease, smoking, common cold within two weeks prior to the operation, previous traumatic intubation history, removable dentures, any congenital or acquired deformity in face, neck, mouth and airway, any known pathology in mouth like aphthous and mouth ulcer,pregnant women, and patients with irregular cycles, and those taking oral contraceptive pills were excluded. By the same protocol general anesthesia was provided and the patients were asked to fill out a three-point scale questionnaire (Low, High, None) 1,6 and 24 hours following intubation to study and record the incidence and severity of sore throat, dysphagia and hoarseness. The date of last menstrual period had been recorded as well.

**Results:**

Of 100 patients, in the first six hours, 51 patients had sore throat and 49 had no pain. During the first 6 hours, 33 patients (33%) had dysphagia and 13 patients had hoarseness at 6th postoperative hour. Age, weight, LMP, intubation time, operation and extubation time and coughing were compared to sore throat, dysphagia and hoarseness. The association between the incidence of coughing and bucking and sore throat was significant (P = 0.03). None of the parameters had a statistically meaningful association with dysphagia.

**Conclusions:**

According to our results, by omitting probable risk factors of incidence of sore throat and evaluation of role of hormonal changes in women represented in menstrual cycles, there was no significant association between menstrual cycle and sore throat incidence.

## 1. Background

Direct laryngoscopy using curved Macintosh blade is the standard technique for facilitating tracheal intubation during general anesthesia (1-3). Providing an appropriate view of the glottis requires applying some force during laryngoscopy and intubation. Therefore, damage to lips, teeth, pharynx, epiglottis, larynx, vocal cords and trachea after managing the airway can be expected, and this may lead to higher rates of morbidity and even mortality in severe cases. Postoperative sore throat is one of the most common complications of endotracheal intubation following operations requiring general anesthesia and prescription of muscle relaxants to facilitate the operation. The prevalence rate of throat complaints is reported at about 40% (14-75%) (3-14). The prevalence of postoperative sore throat is 18%-65% in different studies (6, 15-20). Sore throat and hoarseness along with nausea and pain are among the most common complaints of patients after intubation (3-14). Although throat complaints are often considered as minor complications, they are stressful for patients and usually reminded as a bad memory (6). Several risk factors have been mentioned including female gender (3, 7-9, 21-23), postoperative nausea and vomiting, history of smoking, number of attempts for intubation (6, 9), extubation time, incidence of cough, and straining against the tube, duration of anesthesia, gag, endotracheal tube size, dentures, tracheal tube cuff pressure, presence of blood on tracheal tube (tissue trauma), history of pulmonary disease and so on (6). Other factors like intraoperative position of patients, displacement of the tube during positioning or surgery, etc. may indirectly cause tissue damages in intubation. As we know, menstrual cycle in women is accompanied by a lot of hormonal fluctuations. In the follicular phase, estrogen and FSH levels are increasing; LH abruptly increases during ovulation and in the luteal phase, preference of progesterone is observed. These hormonal fluctuations may affect the quality of life in some women and represent as mood and emotional changes called premenstrual syndrome (PMS). However, the said changes might affect the threshold of pain tolerance. Human studies show increasing sensitivity to pain during the luteal phase for most painful stimuli except electrical stimulation (24). Moreover, response to opioid and nonopioid analgesics is different in women and they require relatively higher doses than men (24-31).

Studies show that gender-related factors like gonadal hormones affect nociceptive processing and analgesic responses. Moreover, gender-related factors merely represent a series of variables that can influence responses to pain and the effect of other uncontrollable factors like age, and psychosocial status can hide gender-related differences. Magnitude and effect of the clinical impact of hormonal influences are not determined. Hormonal effects on responses to pain are naturally cyclic; so, time is an important variable (32, 33).

## 2. Objectives

Considering the above mentioned factors and the point that sore throat is more common among women, in this study, we primarily evaluated the effect of hormonal changes; represented in form of ovulation and endometrial shedding, on pain perception, and the association between the incidence of sore throat and menstrual cycles.

## 3. Patients and Methods

100 non pregnant patients candidate for surgery with general anesthesia age ranged between 18 and 45 were entered the study with the following inclusion criteria consequently. Women of 18-45 years with ASA class I or II without airway problem prediction in clinical examinations (Mallampati 1 and 2 with proper neck movements and three-finger-width mouth opening and the minimum thyromental distance of 6 cm) were selected for operation in the supine position. There should have been no head and neck disposition during the operation and the operation should have been on areas other than the endotracheal tube path including mouth, pharynx, nose, sinus and neck. The possibility of intubation at the end of operation was low. The operations should have not taken more than three hours; the patients should have signed their informed consent and had regular menstrual cycles. The procedures in which we performed in this study included lower abdominal surgery and external ear surgery (mastoidectomy). The exclusion criteria were currently diagnosed pulmonary disease, smoking, common cold within two weeks prior to the operation, previous traumatic intubation history, removable dentures, any congenital or acquired deformity in face, neck, mouth and airway, any known pathology in mouth like aphthous and mouth ulcer, thorax and neck surgeries, operations longer than three hours, pregnancy, taking any oral contraceptive pills,irregular menstrual cycles, and unwillingness to take part in the study.Following the selection and sequential enrollment of the patients in the study, their last menstrual period (LMP), demographic information, and the date of operation were recorded. Then, the patients were provided with their predetermined drug protocol for general anesthesia induction. Basic standard monitoring including ECG, NIBP, pulseoximetry, capnography and temperature monitoring were applied.

**Figure 1.**
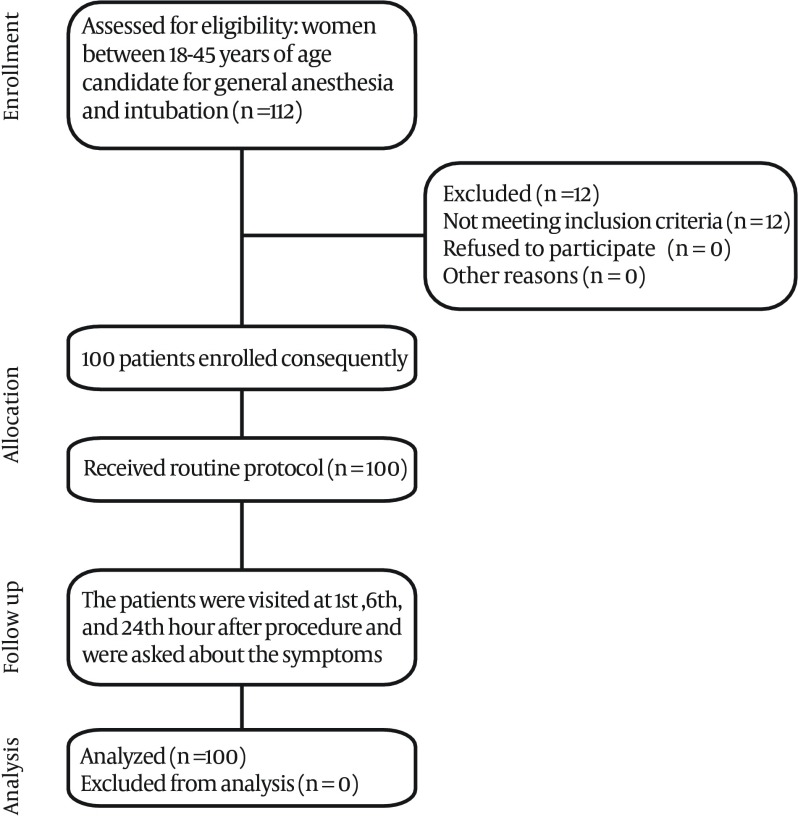
Simple Diagram Demonstrating How Evaluation of Sore Throat was Performed

In the beginning, midazolam 0.03 mg/kg and fentanyl 3μg/kg were prescribed; after two minutes, sodium thiopental 4 mg/kg, lidocaine 1.5 mg/kg and atracurium 0.5 mg/kg were ordered. After three minutes, on minute 5, the patient was intubated by an experienced specialist (attending physician) with globular cuff endotracheal tube No. 7.Ensuring that the endotracheal tube was appropriately placed, the cuff was inflated by tube cuff manometer and the pressure was set between 20-25 mmHg. Anesthesia was maintained by isoflurane 1.2 -2%, oxygen, fentanyl 50-100 μ/h and atracurium 30 mg/h. No lubricant gel or lidocaine spray was used on the endotracheal tube cuff and to prevent possible tracheal damages, unnecessary suction and manipulations were avoided. After securing the tracheal tube, the patient was connected to an anesthesia machine ventilated by TV 10ml/kg, RR 12 without peep.About 20 minutes before the termination of the procedure, ondansetron 4mg was administered intravenously. After discontinuing hypnotic drugs (isoflurane) and with train of four neuromuscular monitoring and at least two responses, neostigmine 0.05mg/kg and atropine 0.02 mg/kg were administered. Following the return of spontaneous breathing and swallowing reflex, the patients were extubated after gentle suction of secretions. In case the patient was coughing and bucking, extubation was postponed to her full awakening and obeying orders. The time interval between discontinuing the anesthetics, extubation and duration of operation were recorded. During anesthesia, fentanyl 50 μg was prescribed every 30-60 minutes so that all patients could have received fentanyl 30 minutes prior to their extubation.The patients were asked to fill out a three-point scale questionnaire (Low, High, None) 1,6 and 24 hours following intubation to study and record the incidence and severity of sore throat, dysphagia and hoarseness. The data was analyzed using SPSS Version 18 statistical software; the quantitative and qualitative data were described as mean (± standard deviation) and ratio, percentage, respectively. According to the type of data, chi 2, t-test and correlation were used in bivariate analyses. Figure 1 demonstrates simply how the study performed. This study was performed without any financial support, and had no additional cost other than usual costs for hospitalization for patients’ procedure. 

## 4. Results

The demographic and primary data of patients and procedure process is depicted in Table 1. 

The incidence of sore throat and dysphagia and hoarseness in 1, 6 and 24 hours after intubation is depicted in Table 2. In the first six hours, 51 patients had pain and 49 had no pain. 97 of 100 patients were in ASA Class I and 3 in ASA Class II. 

78, 19 and 3 patients were intubated on the first, second and third attempt, respectively.

•The number of patients on their 6th or 7th day of menstrual cycle was more (total of 14).

•48 patients were in their progesterone peak (14-28) and 52 were not; however, this did not have any association with pain (P = 0.24).

•During the first 6 hours, 33 patients (33%) had dysphagia; however, this did not have any association with menstrual cycle (P = 0.40).

•Hoarseness had no association with menstrual cycle (P: 0.56).

•Despite receiving prophylaxis, 19 patients had nausea and 6 patients vomited. Among these 19, four patients had sore throat during the first six postoperative hours, but there was no significant association between sore throat and incidence of nausea and vomiting (P: 0.14).

•Age, weight, LMP, intubation time, operation, and extubation time and coughing were compared to sore throat, dysphagia and hoarseness. The association between the incidence of coughing and bucking and sore throat was significant (Binary Logistic Regression P = 0.03)

•None of the parameters had a statistically meaningful association with dysphagia (Binary Logistic Regression P > 0.05)

**Table 1.** Demographic Results and Primary Data of Processes

	Data
**Age, y, Mean ± SD**	31.6 ± 8.4
**Weight, kg, Mean ± SD**	68.6 ± 13
**The interval between LMP and surgery day, Mean ± SD**	14.6 ± 8.4
**Thyromental distancecm, Mean ± SD**	6.5 ± 1.2
**Mallampati range**	1.5(1-4)
**Intubation time, sec, Mean ± SD**	14.3±8.8
**Operation time, min, Mean ± SD**	110±38.3
**Extubation time, min, Mean ± SD**	13.5±6.3
**Cough or bucking or gag (%)**	34

**Table 2.** Incidence of Sore Throat

	1^st^ hour (%)	6^th^ hour (%)	24 hour (%)
**Mild sore throat**	34	32	15
**Sever sore throat**	7	4	1
**Dysphagia **	30	17	11
**Hoarseness **	18	13	9

## 5. Discussion

**Figure 2.**
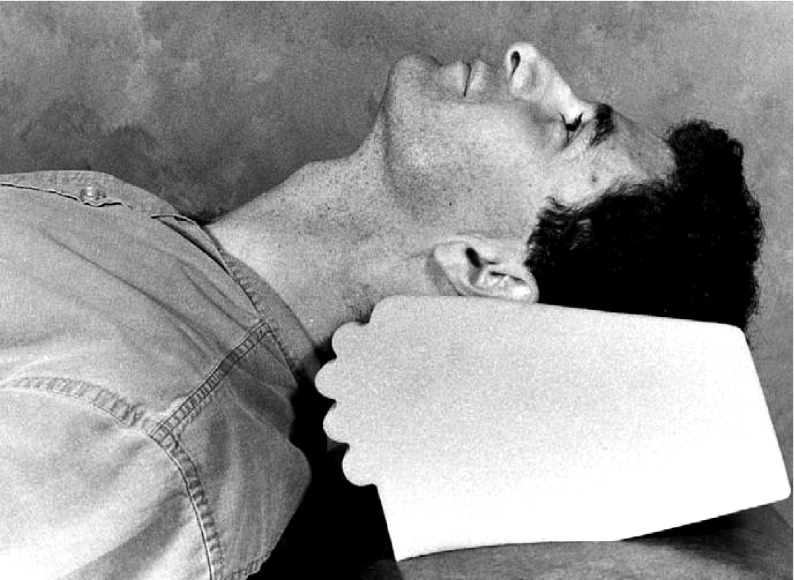
Sniff Position for Better Laryngoscopy

**Figure 3.**
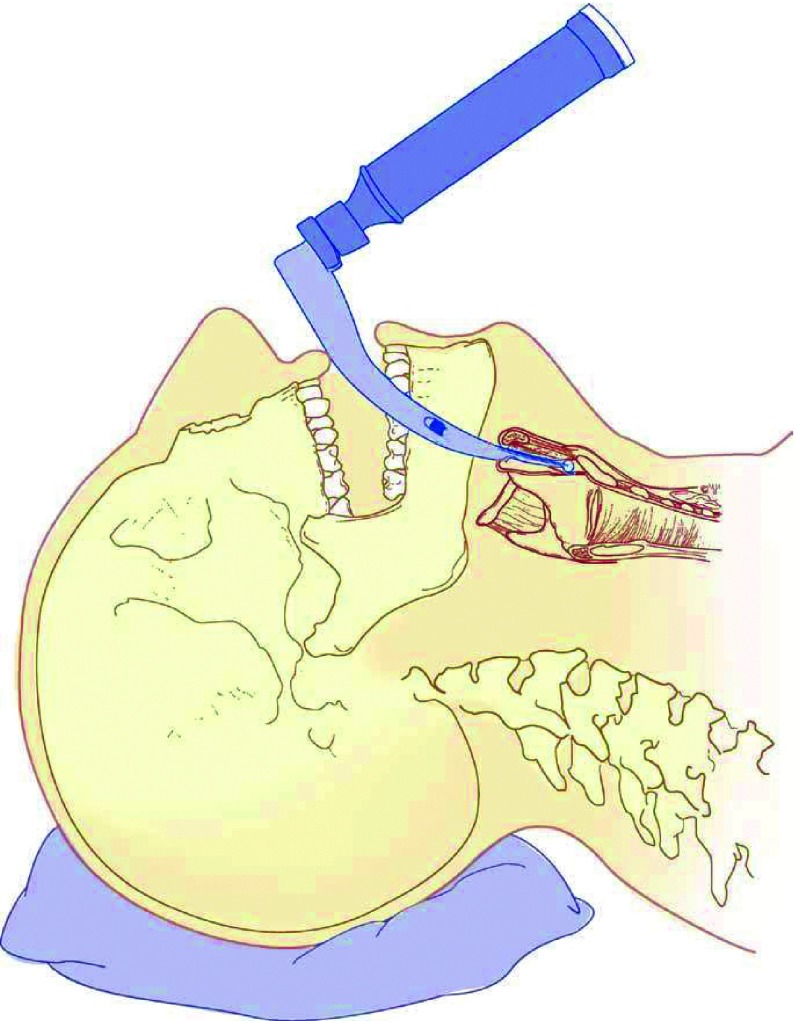
Laryngoscopy; Note the Position of Blade tip in Front of Epiglottis

According to our results, by omitting probable risk factors of incidence of sore throat and evaluation of role of hormonal changes in women represented in menstrual cycles, there was no significant association between menstrual cycle and sore throat incidence; however, the incidence of sore throat was significantly related to inherent and technical factors related to anesthesia including coughing and straining against tracheal tube and extubation time (P< 0.05). The patients were extubated after administering nausea and vomiting prophylaxis (ondansetron).In their clinical examination, all patients were found with relatively simple airways and were intubated with blade 3 mackintosh; they fell into Mallampati 1 or 2 classes. In the present study, procedures of maximum 3 hours without major dispositions were considered. There is minimum disposition of head, neck and endotracheal tube in gynecologic, external ear and lower abdominal procedures. Hence, such procedures were examined in this study to reduce unwanted trauma during disposition. To equalize the impact of tube size, all patients were intubated by globular cuff tube No.7. All tubes were the same type and were fixed opposite maxillary central incisors. Cuff pressure was maintained at 20-25 mmHg by a manometer. Thus, attempts were made to omit possible causes of sore throat to focus on the pure impact of menstrual cycle (and hormonal status) on the incidence of sore throat.Performing successful direct laryngoscopy depends on achieving a line of sight from the maxillary teeth to larynx. Patients are put in sniff position for direct laryngoscopy (Figure 2). Using a laryngoscope blade, epiglottis and tongue are removed from the line of sight; tongue is usually moved to the left horizontally. Hyoid bone and the connective tissue are moved to the front, the epiglottis is projected either directly or indirectly, and the larynx could be seen. The force applied to the laryngoscope handle should be strong enough to project hyoid bone and the connective structures parallel to the line of sight ( 34 , 35 ). Sufficient projecting force whichmight cause considerable tissue damage is a key factor in successful direct laryngoscopy (Figure 3). Achieving the best laryngeal view without causing tissue trauma is significant; however, reaching a line of sight with laryngoscopy is not always possible. Direct laryngoscopy using Macintosh curved blades is the standard technique for facilitating tracheal intubation during anesthesia; however, this method has some limitations ( 1 - 3 ). First, learning the skill is not easy. Second, unifying the oropharyngolaryngeal axis is not always possible ( 2 , 36 ). Third, routine clinical tests are not powerful enough in predicting such problems ( 2 , 37 ). Therefore, even most expert physicians would face difficulties in 1.5%-8.5% of laryngoscopy cases (Cormack & Lehane Class 3-4) ( 2 ). Despite its unquestionable advantages like preventing aspiration, reducing airway dead space for suction and controlled ventilation, tracheal intubation may cause some complications ( 6 ). Providing an appropriate subglottic view requires applying force through the process of laryngoscopy and intubation; thus, damage to lips, teeth, throat, epiglottis, larynx, vocal cords and trachea after managing the airway is predictable and this leads to higher morbidity rates and even mortality in severe cases. Moreover, beside intubation there are other factors like the patient's position during the operation, moving the tube while changing the patient's position or the type of operation which can indirectly cause tissue damages. The prevalence of postoperative sore throat varies from 18% to 65% in different studies ( 6 , 15 - 20 ); however, it is not clearly known if there is an association between the incidence of postoperative sore throat and menstrual cycles. Different studies have focused on facilitating intubation and comfort in using laryngoscopes and more recent instruments like glidescope more and some of these studies are performed on dummies or difficult-airway simulated models ( 1 , 2 , 20 , 38 - 42 ). During the first six hours after extubation, 51 patients had pain and 49 did not (51%); this data is compatible with the existing studies ( 3 - 14 ). The interesting point is that the number of patients in their sixth or seventh menstrual cycle was more and this does not have a specific justification; yet, perhaps it can be said that women were free from menstrual bleeding during this time and they could comfortably refer for elective surgeries. Nevertheless, this did not have any association with the incidence of sore throat. 

**Figure 4.**
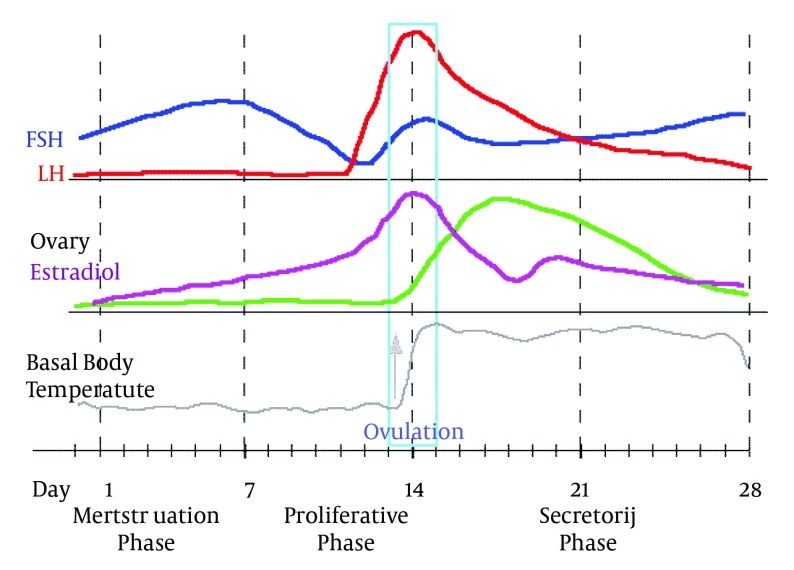
Hormonal Changes During Menstrual Period

Ovulation physiologically happens in women every month and in the absence of conception and implantation in uterus, the endometrium naturally sheds and after the termination of bleeding, the next cycle begins. The first menses day is the first day of the monthly cycle and the mean duration of menses is5±2 days. The average duration of monthly cycles is 28 days (ranging from 25 to 36 days, while 20-45 days is also reported in some women). Monthly cycles can be divided into three phases including the follicular phase during which estrogen and FSH levels are increasing; the ovulation phase in which a series of complicated endocrine events lead to LH surge, and the luteal phase with progesterone preference which follows ovulation. The luteal phase is 14 days and terminates on the first menses day (Figure 4) ( 43 ). Variations incycles duration are related to follicular phase variations; hence, using the information on LMP (Last Menstrual Period) or the first day of the last menstruation, the approximate time of FSH, LH, progesterone peak and ovulation could be estimated. These hormonal fluctuations called premenstrual syndrome (PMS), affect the quality of life of women in form of mood and emotional changes. These hormonal changes might even influence the threshold of pain tolerance. In a study conducted on pain tolerance threshold, it was revealed that higher threshold of pain and more tolerances are observed for most painful stimuli except electrical stimulation during the follicular phase compared to luteal and periovulatory phases, and the amount of impact is mild to moderate. Unfortunately, serum gonadal levels required for confirming ovulation and determining the association between hormonal activities and pain responses are not taken into consideration in most studies. Generally, human studies show increasing sensitivity to pain during the luteal phase for most painful stimuli except electrical stimulation ( 24 ). Also, response to opioid and nonopioid analgesics is different in women and they require relatively higher doses than men ( 24 - 31 ) but unfortunately human studies have not specified the effect of cycles or administration of exogenous hormones on analgesic responses. Overall, the studies show that gender-related factors like gonadal hormones affect nociceptive processing and analgesic responses. Moreover, gender-related factors merely represent a series of variables which can influence responses to pain, and the effect of other uncontrollable factors like age and psychosocial status can hide gender-related differences. Magnitude and the clinical impact of hormonal influences are not determined. Hormonal effects on responses to pain are naturally cyclic; so, time is an important variable ( 39 , 42 ).There were 48 patients in peak levels of progesterone (days 14-28) but this was not related to pain. In fact, there is no difference or if there is, it might be so slight that the effect of cycles is not evident due to known or unknown factors, individual differences, differences between surgeons and so on. 

During the first six hours, 33 patients complained about dysphagia but no association with a menstrual cycle was observed.During the first six hours, 20 patients complained about hoarseness but it was not related to menstrual cycles. Age, weight, LMP, intubation time, duration of operation, extubation time and incidence of coughing and bucking during extubation were compared to sore throat; the incidence of sore throat has a significant association with coughing (Binary Logistic Regression, P = 0.03), and hoarseness was associated with coughing and extubation time (Binary Logistic Regression, P = 0.014 and 0.031, respectively). During emergence stages, the patients were coughing and bucking and we tried to avoid the discomfort by deep extubation. This happened to some patients before extubation and it led to sore throat and voice hoarseness. Since the glottal opening narrows during coughing and bucking due to contractions of laryngeal muscles and vocal cords come together, traumatization happens despite having an endotracheal tube and the symptoms present after extubation.Furthermore, the longer the extubation time, the more severe the hoarseness and this shows a higher possibility of coughing and bucking on the tube during emergence and consequently vocal cord trauma. Our inability to check hormone levels due to lack of budget was a limitation for us to perform more accurately. So we asked them the LMP date and calculated the day of cycle which they were in. It can be concluded from the study that hormonal changes and consequently menstrual cycles do not spontaneously affect the incidence of post extubation sore throat, but technical and natural intubation issues, extubation and airway traumatization are more effective.

The incidence of sore throat within the first six hours after extubation is one of the factors affecting both patient satisfaction and surgical outcome because complications like heart rate, blood pressure, bleeding, opioid consumption and their side effects (nausea, apnea) increase with pain. So, the authors recommend the modification of intubation techniques, providing more care during extubation, so that the patient does not cough and buck. Application of methods like Lidocaine IV for suppressing laryngeal reflexes before extubation and minimizing innate endotracheal tube trauma to reduce patients' pain and complications of intubation. As a result, the quality of life of patients and their satisfaction would be increased and the duration of hospitalization as well as the resulting complications and costs would be decreased.
